# Effectiveness of aerobic and resistance training on the motor symptoms in Parkinson's disease: Systematic review and network meta-analysis

**DOI:** 10.3389/fnagi.2022.935176

**Published:** 2022-08-01

**Authors:** Xiao Zhou, Peng Zhao, Xuanhui Guo, Jialin Wang, Ruirui Wang

**Affiliations:** ^1^Sports Rehabilitation Research Center, China Institute of Sport Science, Beijing, China; ^2^College of Sport Medicine and Rehabilitation, Beijing Sport University, Beijing, China

**Keywords:** Parkinson's disease, aerobic exercise, resistance exercise, intervention, network meta-analysis

## Abstract

**Background/objectives:**

Aerobic and resistance training are common complementary therapies to improve motor symptoms in people with Parkinson's disease (PD), and there is still a lack of advice on which intensity and period of aerobic or resistance training is more appropriate for people with PD. Therefore, a network meta-analysis was conducted to assess the comparative efficacy of aerobic and resistance training of different intensities and cycles on motor symptoms in patients with Parkinson's disease.

**Methods:**

Based on several biomedical databases, a search strategy system was conducted to retrieve randomized controlled trials (RCTs) without language restrictions. A network meta-analysis with a frequentist approach was conducted to estimate the efficacy and probability rankings of aerobic and resistance training on Parkinson's patients. What's more, a range of analyses and assessments, such as routine meta-analyses and risk of bias, were performed as well.

**Results:**

Twenty trials with 719 patients evaluating 18 different therapies were identified. Through the Unified Parkinson's Disease Motor Rating Scale, (UPDRS III); 6-minute walk test, (6MWT); 10-meter walk test, (TWM); and time up and go (TUG) and Quality of Life Scale-39 (PDQ-39), to explore the effects of different intensity resistance and aerobic exercise on PD. As a result, short period high intensity resistance movement (standard mean difference (SMD) = −0.95, 95% confidence interval (CI) −1.68 to −0.22) had significantly decreased the Unified Parkinson's Disease Motor Rating Scale (UPDRS III). Short period high intensity resistance exercise showed similar superiority in other indices; also, aerobic and resistance training of different cycle intensities produced some efficacy in PD patients, both in direct and indirect comparisons.

**Conclusion:**

For patients with moderate to mild Parkinson's symptoms, short periods high intensity resistance training may provide complementary therapy for PD, and aerobic or resistance training of varying intensity and periodicity may be recommended as exercise prescription for PD patients. However, more large scale and high quality clinical trials are needed to confirm the effectiveness of this exercise therapy in the future.

**Systematic Review Registration:**

https://www.crd.york.ac.uk/PROSPERO/, identifier: CRD42022324824.

## Introduction

Parkinson's disease (PD) is the second most common neurodegenerative disease whose prevalence is projected to double over the next 30 years and affect ~611 million people worldwide (Dorsey et al., 2018; Armstrong and Okun, 2020; Bloem et al., 2021; Tolosa et al., 2021). The histopathology of PD is typically a loss of dopaminergic neurons in the substantia Nigra, and the cardinal features of PD include resting tremor, rigidity, and bradykinesia. Apart from the above symptoms, people with PD also suffer from various non-motor features such as sleep disorders, psychiatric symptoms, and cognitive dysfunction (Armstrong and Okun, 2020). Currently, no therapy can slow down or arrest the progression of PD (Bloem et al., 2021). Meanwhile, the number of patients with PD has more than doubled in the past generation due to an increase in the elderly population (2018), As a consequence, PD poses a significant challenge for the global health systems that must be resolved.

With no known cure for PD, Levodopa remains the most effective first-line treatment for motor symptoms in PD (Elkouzi et al., 2019; Armstrong and Okun, 2020; Koszła et al., 2021). Despite the effectiveness of pharmacotherapy, serious deficiencies in the long-term treatment, namely, the induction of dyskinesia and drug fade, cannot be ignored (Duncker and Bache, 2008). To avoid the negative effects of levodopa, exercise therapy, as a low-cost and universally available aid, is adopted in the current PD treatment. There is growing evidence that exercise therapy is beneficial as a complementary therapy to improve motor and non-motor symptoms such as slow movement, decreased muscle strength and reduced quality of life in PD patients when mainline medications fail to respond appropriately to the motor symptoms of PD (Speelman et al., 2011; Mak et al., 2017; Mak and Wong-Yu, 2019). Among all the common types of exercise, aerobic and strength training, recommended by the World Health Organization (WTO), play a crucial role in improving exercise performance and slowing the progression of PD (World Health Organization, 2010; Speelman et al., 2011; Carvalho et al., 2015; Mak et al., 2017; Mak and Wong-Yu, 2019). It is worth mentioning that in recent years, different evidence has emphasized the therapeutic potential of aerobic and resistance exercise for PD (Dutra et al., 2012; Churchill et al., 2017; Gamborg et al., 2022). In a major review, Petzinger et al. (2013) concluded that Aerobic exercise, regarded as important for improvement of blood flow and facilitation of neuroplasticity in elderly people, might also have a role in improvement of behavioral function in individuals with PD. In a recent systematic review of five randomized controlled trials (RCT), resistance exercise was found to have a positive effect on muscle strength, mobility, endurance and performance in functional tasks (Dibble et al., 2006, 2009b; Morris et al., 2017; de Lima et al., 2019). Furthermore, some studies have shown that resistance training can improve strength, balance and improve the quality of life of people affected by PD (Dibble et al., 2009b).

However, other research presented different results in terms of the positive effects of resistance training. In a meta-analysis including six studies and conducted by Saltychev et al. (2016). It was found that resistance training was not superior to other treatment modalities for PD. In another research, Morris et al. (2017) performed a 6-week home resistance exercise program and concluded that it did not improve falls in 133 community-dwelling PD patients. In addition to the controversial results of resistance training studies, previous reviews of meta analyses of aerobic or resistance training for PD did not classify exercise dose (e.g., exercise intensity, exercise cycle, exercise type, etc.) (Chung et al., 2016; Lavin et al., 2020), which is necessary for exercise prescription in practice. No meta-analysis has compared any types of aerobic exercise, resistance exercise, or a combination of both. With the above deficiencies in resistance training research, thus, we attempted to re-evaluate the effect of aerobic exercise and resistance training on PD through a net-work meta-analysis. By means of dividing the exercise intensity and the exercise period by the proposed ACSM (Riebe et al., 2015). we further compared the effects of different intensity and periods of aerobic and resistance training on motor symptoms in Parkinson's patients in the hope of providing a low cost and useful exercise program for PD.

## Materials and methods

### Study registration

The systematic review protocol was developed on the strength/ basis of the Preferred Reporting Items for Systematic Review and Meta-Analysis Protocols (PRISMA-NMA) statement (Moher et al., 2015) registered in the PROSPERO database. This systematic review was registered in advance in the PROSPERO register of systematic reviews (ref: CRD42022324824). Since all the analyses were based on previously published research, there was no need for ethical approval or patient permission.

### Search strategy and study selection

**Figure 1:**
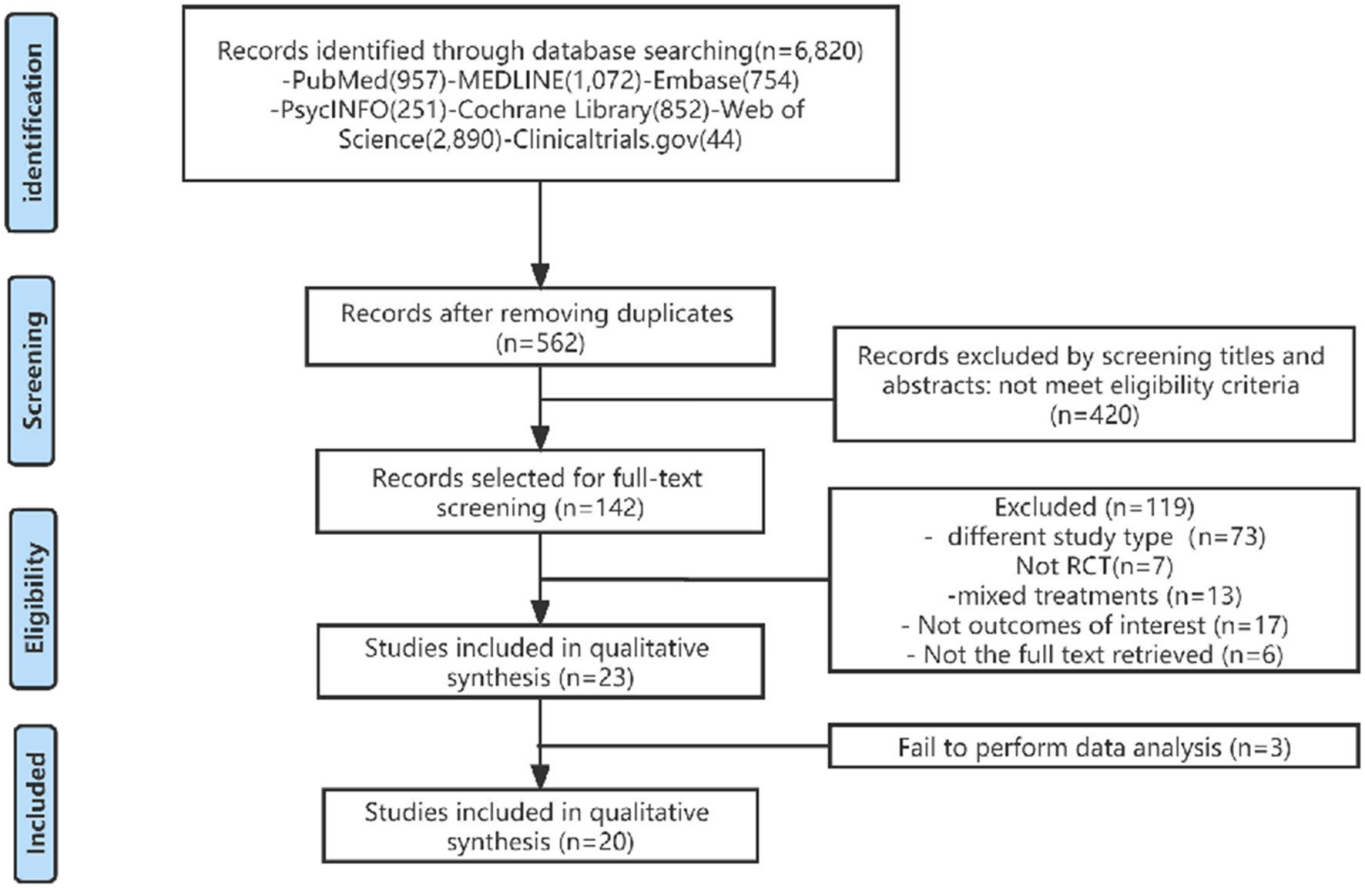
Flow chart illustrating the different phases of the search and study selection.

The following electronic databases were searched from their inception to April 2022: PubMed, EMBASE, PsycINFO, Cochrane Library, ClinicalTrials.gov, and Web of Science without language restrictions. Targeted at studies published between the start of the project and the date of the search, the retrieval was rerun shortly before the final analysis, with additional studies retrieved for inclusion (Briscoe, 2018; Cooper et al., 2018). We adopted a Boolean search strategy with the operators AND, OR, and NO as well as terms describing or relating to intervention, participants, and study design. The Medical Subject Headings (MeSH) combined with text terms followed by Boolean logical operators were used as an exhaustive search using “Parkinson's disease,” “exercise,” “resistance training,” “aerobic exercise,” “bodyweight support treadmill,” “high speed resistance training,” “Nordic Walking,” “power training,” “treadmill training,” “walking,” “Randomized controlled trials” and other relevant conceptual keywords. We used various combinations of medical subject headings and free terms, such as Parkinson's disease and aerobic and resistance exercise. A flowchart describing the literature selection is presented in Figure 1. In addition, reference lists were hand searched to identify further relevant articles (Supplementary material 1).

All search results were exported into EndNote and duplicates were removed. Titles and abstracts from the initial literature search were independently assessed by two reviewers (ZX and WJL). Full texts for articles were deemed eligible for inclusion from the title and abstract search by either reviewer; in addition, when opinions differed in an article in the initial screen, it would be screened independently a second time by two researchers (GXH and WRR).

### Inclusion and exclusion criteria

Based on the defined inclusion and exclusion criteria (Table 1): (1) All studies were randomized controlled trials (RCTs). (2) The population of the included studies was adult patients diagnosed with PD and Hoehn and Yahr scale (H&Y): 1-3. (3) Interventions included at least one of the following exercises: Aerobic exercise; Treadmill training; Nordic Walking and Multicomponent exercise program; Resistance exercise: Power training; Strength Training; Weight Lifting Exercise Program. The control group received Usual care. (4) Outcomes: (1) Motor outcomes: Motor ability was evaluated by the Unified Parkinson's Disease Motor Rating Scale, UPDRS III; walking ability was evaluated by a 6-minute walk test, 6MWT; 10-meter walk test, TWM; and time up and go, TUG. (2) Non-motor outcomes: quality of life was evaluated by the Parkinson's Disease Quality of Life Scale-39 (PDQ-39); studies were excluded if insufficient data or in-formation about the assessment was provided. We also excluded quasi-RCTs, animal trials, clinical protocols, meeting abstracts, case reports, and systematic reviews.

**Table 1:** Selection criteria.

**Category**	**Inclusion criteria**	**Exclusion criteria**
Population	Aged 20–85 years;diagnosed with Idiopathic Parkinson's (IPD) and Hoehn and Yahr scale (H&Y).	Other specific diseases
Intervention	Aerobic exercise; Treadmill training; Nordic Walking and Multi component exercise program (Aerobic exercise combined with resistance exercise or multiple aerobic exercises). Resistance exercise: Power training; Strength Training; Weight Lifting Exercise Program; Multi component exercise program (Aerobic exercise combined with resistance exercise or multiple resistance exercises).	Taichi, Qigong and other physical and mental exercises; dance training; stretching, balance; flexibility training; multimodal training.
Comparator	There is only a passive control (i.e., without any regular training) that allows for normal drug taking.	Other types of active exercises (tai chi, qigong and other mind-body exercises mentioned above; dance training; balance training; flexibility training; multimodal training, etc.)
Outcome	Unified Parkinson's Disease Motor Rating Scale, UPDRS III; walking ability was evaluated by a 6-minute walk test, 6MWT; 10-meter walk test, TWM; and time up and go, TUG. Parkinson's Disease Quality of Life Scale-39 (PDQ-39)	Strength training indicators: muscle strength, muscle hypertrophy, etc.; neurological test-related indicators, etc.
Study design	Randomized controlled trials	Quasi RCTs, animal trials, clinical protocols, meeting abstracts, case reports, and systematic reviews.

### Outcome measurement

Extracted from the final inclusion list of articles separately by two reviewers the data was then included into a standardized data extraction spreadsheet in Excel. At this stage, two authors extracted information on (1) relevant data regarding participant characteristics (e.g., the sample size, age, and sex); (2) the training pattern; (3) the training variables (e.g., duration, repetitions, and intensity); (4) years of diagnosis; (5) Hoehn and Yahr scale (H&Y); (6) the main result of the study. In case of incomplete raw data availability, we contacted the corresponding author of the manuscript, and studies whose authors could not be reached were left out. Our primary outcome of interest was assessed by the Unified Parkinson's Disease Motor Rating Scale, UPDRS III; other outcomes included a 6-minute walk test (6MWT), 10-meter walk test (TWM), time up and go (TUG) and Parkinson's Disease Quality of Life Scale-39 (PDQ-39).

### Coding of studies

We coded RT according to the following training parameters: training period, training intensity. According to the recommendations of American College of Sports Medicine's (ACSM) (American College of Sports Medicine Position Stand, 1998; Riebe et al., 2015). We further divided aerobic exercise and resistance exercise according to the intensity of exercise and training period. For aerobic exercise, exercises with 30–40% heart rate reserve (HRR); 37–45% maximal oxygen consumption (VO_2max_); 57–63%%Maximal heart rate (HR_max_); 9–11 rate of perceived exertion (RPE) or 2–3 metabolic equivalent (MET) were defined as low intensity aerobic exercise. Exercises with 40–59%HRR; 46–63%VO_2max_; 64–75%HR_max_; RPE 12–13 or MET 3–6 were defined as moderate intensity aerobic exercise. Exercises with 60–90% HRR; 64–91% VO_2max_; 76–95% HR_max_; RPE 14–17 or MET 6–8.8 were defined as high intensity aerobic exercise.

For resistance training, exercises with <50% one-repetition maximum (1RM) or 8–12 repetitions exercise is defined as low intensity resistance training. 50–69% 1RM or 10–15 repetitions exercise is defined as moderate intensity resistance training. >70% 1RM or 15–20 repetitions exercise is defined as high intensity resistance training. If a study reported exercise progression over the training period, the mean number of training intensity was computed. The definition of the exercise period in the previous literature is Lacking, and based on clinical experience. Exercises with a training period greater than 12 weeks are defined as long period exercises, those with a training period of 6–12 weeks are defined as medium period exercises, and those with a training period of less than 6 weeks are defined as short period exercises.

Therefore, we coded exercise according to the following training parameters: there are the following 18 types of exercise, respectively: short period low intensity aerobic exercise (SP-LI-AE), short peri-od moderate intensity aerobic exercise (SP-MI-AE), short period high intensity aerobic exercise (SP-HI-AE), short period low intensity resistance training (SP-LI-RT), short period moderate intensity resistance training (SP-MI-RT), short period high intensity resistance training (SP-HI-RT), moderate period low intensity aerobic exercise (MP-LI-AE), moderate period moderate intensity aerobic exercise (MP-MI-AE), moderate period high intensity aerobic exercise (MP-HI-AE), moderate period low intensity resistance training (MP-LI-RT), moderate period moderate intensity resistance training (MP-MI-RT), moderate period high intensity resistance training (MP-HI-RT), long period low intensity aerobic exercise (LP-LI-AE), long period moderate intensity aerobic exercise (LP-MI-AE), long period high intensity aerobic exercise (LP-HI-AE), long period low intensity resistance training (LP-LI-RT), long period moderate intensity resistance training (LP-MI-RT), long period high intensity resistance training (LP-HI-RT) (Table 2).

**Table 2:** The movement characteristics and classification principles.

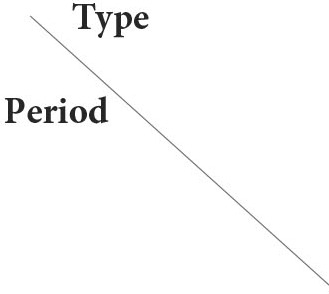	**Low intensity aerobic exercise (30–40% HRR; 37%-45% VO_2max_; 57–63%% HR_max_; 9–11 RPE; 2–3 MET)**	**Moderate intensity aerobic exercise (40–59%HRR; 46–63%VO_2max_; 64–75%HRmax; RPE 12–13 or MET 3–6)**	**High intensity aerobic exercise (60–90% HRR; 64–91% VO_2max_; 76–95% HRmax; RPE 14–17 or MET 6–8.8)**	**Low intensity resistance training (<50% 1RM or 8–12 repetitions exercise)**	**Moderate intensity resistance training (50–69% 1RM or 10–15 repetitions)**	**High intensity resistance training (>70% 1RM or 15–20 repetitions)**
Short period exercises (<6 weeks)	Short period low intensity aerobic exercise	Short period moderate intensity aerobic exercise	Short period high intensity aerobic exercise	Short period low intensity resistance training	Short period moderate intensity resistance training	Short period high intensity resistance training
Moderate period exercises (6–12 weeks)	Moderate period low intensity aerobic exercise	Moderate period moderate intensity aerobic exercise	Moderate period high intensity aerobic exercise	Moderate period low intensity resistance training	Moderate period moderate intensity resistance training	Moderate period high intensity resistance training
Long period exercises (>12 weeks)	Long period low intensity aerobic exercise	Long period moderate intensity aerobic exercise	Long period high intensity aerobic exercise	Long period low intensity resistance training	Long period moderate intensity resistance training	Long period high intensity resistance training

*HRR, heart rate reserve; VO_2max_, maximal oxygen consumption; HR_max_, 57–63% Maximal heart rate; RPE, rate of perceived exertion; MET, metabolic equivalent; 1RM, one-repetition maximum*.

### Risk of bias (quality) assessment

We assessed and classified each individual's risk of bias (ROB). Studies were first selected according to the Cochrane Risk of Bias tool (Tarsilla, 2010) and then classified according to priority criteria Seven. ROB domains were evaluated independently. Two authors categorized all the eligible studies as high, low, or indifferent uncertain bias risk, including random sequence generation, allocation concealment, blinding of participants and personnel, blinding of outcome assessment, incomplete outcome data, selective reporting, and other biases. The evaluation of ROB was carried out in Review Manager (Version 5.3). In the selective outcome data, we accounted for a broader assessment considering also the selective non reporting ROB due to the missing results in index meta analyses (e.g., missing or unavailable outcome results crosschecked from method plans) according to published criteria by Page et al. For each study, the items were scored as high, low or unclear (not enough information reported) ROB. Two reviewers scored the studies according to the proposed scale. In case of disagreements on the scores, a consensus was adopted; if necessary, a third reviewer evaluated the article (WRR).

### Data synthesis and analysis

The network meta-analysis has a categorical advantage over traditional meta-analysis due to its ability to summarize comparisons between multifarious treatments concurrently, which allows greater flexibility to use complex models and produces relatively scientific interpretation in terms of causal relationships (Stroup et al., 2000). Based on the random effect statistical model, the prior minimally informative distributions were implemented to compare 18 exercise therapies simultaneously by forming a connected network integrating direct and indirect evidence. We first carried out a conventional pairwise meta-analysis in comparisons available for each contrast. In terms of statistical heterogeneity, *I*^2^ statistic whose values were 25%, 50%, and 75% indicated mild, moderate, and high heterogeneity, respectively, and was provided to measure whether there was a substantial heterogeneity generation (Melsen et al., 2014). A comparison adjusted funnel plot was drawn for detecting the presence of any dominant types of bias, such as publication bias, selective reporting, and the like.

A network plot was generated as a simple/ brief summary description for revealing all the available evidence of each treatment evidence. The above analyses were conducted in STATA, version 16.0 (Stata, Corp, College Station, TX). As the presence of effect sizes refers to the continuous outcome, standard mean differences (SMDs) were calculated for each comparison using group (relevant) means and standard deviations (SDs) from individual studies (Hirschtritt et al., 2017). The flowing 95% Confidence Interval (CI) and pooled SMDs were calculated as a measurement of estimated uncertainty and pooled effect sizes, respectively. If the data we were to extract for our analysis (such as mean, SD, or sample size) were not provided in the included literature, we would present them in another form by calculating other available values such as standard errors, confidence intervals, or other statistical indices as described elsewhere which may clarify SD accordingly (Egger et al., 2003).

Network transitivity is the most important assumption underlying NMA, whose assessment would directly affect our further analysis (Salanti, 2012). Therefore, to ensure the similarity of various treatment comparisons so as to provide valid indirect inferences, we apprised the transitivity assumption by comparing the clinical and methodological characteristics such as patients and experimental designs in all the included studies (Tarsilla, 2010). As an estimated probability used for ranking the different exercise types of intervention, the surface under the cumulative ranking curve (SUCRA) was presented as a simple numerical statistic cumulative ranking probability plot summarized for each treatment. SUCRA with a higher value denotes a greater likelihood of a given treatment being in the top rank or highly effective, while zero indicates that the treatment is definitely the worst (Page et al., 2016). To make sure whether a potential source inconsistency will be generated in our network or not, we used the “node-splitting” technique (van Valkenhoef et al., 2016), by comparing the direct evidence to the indirect from the entire network (with a *p*-value higher than 0.05 indicating a consistency generation) (Stang, 2010). The above analyses were per-formed using the “network “and “meta” packages (version 0.8-2) in R language (X64 3.32 version).

## Results

### Study selection

All included trials were published between 2006 and 2022, 20 eligible RCTs with 811 patients diagnosed with PD were included in this network meta-analysis (Dibble et al., 2006, 2009b, 2015; Fisher et al., 2008; Kurtais et al., 2008; Schilling et al., 2010; Canning et al., 2012; Qutubuddin et al., 2013; Shulman et al., 2013; Frazzitta et al., 2014; Paul et al., 2014; Carvalho et al., 2015; Cugusi et al., 2015; Ni et al., 2016; Demonceau et al., 2017; Santos et al., 2017; Ferreira et al., 2018; Schenkman et al., 2018; van der Kolk et al., 2018; Vieira de Moraes Filho et al., 2020) (Supplementary material 2). Four hundred and sixteen participants (49%) were in the control group, and 395 (51%) in the exercise group, most of whom' were diagnosed with mild-moderate PD (Hoehn and Yahr scale I–III), two studies described as IPD (Paul et al., 2014; Dibble et al., 2015). In terms of medication taking, five studies did not mention their medication use (Frazzitta et al., 2014; Paul et al., 2014; Carvalho et al., 2015; Demonceau et al., 2017; Vieira de Moraes Filho et al., 2020), one study (van der Kolk et al., 2018) was tested in the “OFF” phase and the rest of the studies were assessed and tested in the “ON” phase of the patients, the specific literature screening results characteristics of the different types of studies are shown in Table 3.

**Table 3:** The specific movement characteristics and classification principles.

**Publication**	**Inclusion criteria**	**N (E/C)**	**Mean age ±SD ALL**	**Intervention (E/C)**	**Intensity (E/C)**	**Exercise intensity and period categories**	**Frequency (days/wk/ month)**	**Main outcome assessments**			**Pharmacological treatments**
Ferreira et al. (2018)	Hoehn-Yahr 1–3	35 (18/17)	65.9 ± 7.95	Resistance training	IN: 8–12 repetitions	Long period high intensity resistance training)	2/wk/6M	UPDRS	PDQ-39		“on” state of medication
				Usual care	Usual care						Customary medication
Vieira de Moraes Filho et al. (2020)	Hoehn-Yahr 1–3	40 (25/15)	64.6 ± 5.5	Resistance training	IN: 2 sets of 10–12 repetitions until fatigue	Short period high intensity resistance exercise	2/9 wk	TMW	TUG	UPDRS-III	NA
				Disease lectures	Usual care						NA
Santos et al. (2017)	Hoehn-Yahr 1–2	28 (13/15)	73.6 ± 15.86	PRE training	IN: 1 set of 15–20 repetitions at 40–50% of 1RM/1–2 W 2 sets of 7–10 repetitions at 70–75% 1RM/3–6 W 2 sets of 4–7 repetitions at 80-85% of the 1RM/7–8 W	Short period high intensity resistance exercise	2/8 wk	MDS-UPDRS	PDQ-39	TMM	“on” state of medication
				Usual care	Usual care						Customary medication
Schenkman et al. (2018)	Hoehn-Yahr 1–2	128 (43/45/40)	63.7 ± 9.67	Intensity treadmill exercise	IN: 80–85% MHR	Long period high intensity aerobic exercise	4/26 wk	UPDRS	MDS-UPDRS		“on” state of medication
				Moderate intensity treadmill exercise	IN: 60–65% MHR	Long period moderate intensity aerobic exercise					“on” state of medication
				Usual care	Usual care						Customary medication
Ni et al. (2016)	Hoehn-Yahr 1–3	26 (14/10)	73.3 ± 7.45	power resistance training	IN: 3 circuits of 10–12 repetitions	Long period high intensity aerobic exercise	2/12 wk	PDQ-39			“on” state of medication
				Normal group	Usual care						Customary medication
Dibble et al. (2009b)	Hoehn-Yahr 2–3	20 (10/10)	65.7 ± 9.9	Eccentric training	IN: high-force Weight was increased as tolerated	Long period high intensity resistance exercise	3/12 wk	6 MW	UPDRS-III		1–1.5 h after taking their PD medications
				Standard exercises	Usual care						1–1.5 h after taking their PD medications
Dibble et al. (2006)	Hoehn-Yahr 2–3	20 (10/10)	65.7 ± 9.9	Eccentric training	IN: high-force Weight was increased as tolerated	Long period high intensity resistance exercise	3/12 wk	6 MW			1–1.5 h after taking their PD medications
				Standard exercises	Usual care						1–1.5 h after taking their PD medications
Demonceau et al. (2017)	Hoehn-Yahr 1–3	52(20/17/15)	66 ± 9	Aerobic training	IN: High intensity cycling at 70% to 80% 30 s to three min of high intensity cycling at 70% to 80% of PWL	Long period high intensity resistance exercise		TUG	PDQ-39		NA
				Strength training	IN:10–15 repetitions at 50–60% /1RM/1–6W; 5-8 repetitions at 80–90% of the 1RM/7–12 W	Long period high intensity aerobic exercise	2–3/12 wk				NA
				Care, control group	Usual care						Customary medication
Frazzitta et al. (2014)	Hoehn-Yahr 1–1.5	25 (15/10)	NR	Aerobic training	IN: MHR ≤ 60% and a maximum speed of treadmill scrolling of 3.5 km/h. 30 min/20 sessions	Short period low intensity aerobic exercise	20/4 wk	UPDRS-III	6 MWT		NA
				Normal group	Usual care						Customary medication
Canning et al. (2012)	Hoehn-Yahr 1-2	20 (10/10)	61.8 ± 7.9	Home-based treadmill training	IN:60% of the average speed	Short period moderate intensity aerobic exercise	4/6 wk	6 MWT	PDQ-39	TMW	“on” state of medication
				Usual care	Usual care						Customary medication
Shulman et al. (2013)	Hoehn-Yahr 1–3	67 (23/22/22)	65.7 ± 10.83	Higher-Intensity Treadmill Training	IN: 70–80% of HRR (30 min)	Long period high intensity aerobic exercise	3/12 wk	6 MW	TMW	UPDRS -III	“on” or within 3 h of medication
				Lower-Intensity Treadmill Training	IN:15 min 0% incline (start) increased 5 min every 2 weeks at 40–50% of MHR (50 min)	Long period low intensity aerobic exercise					“on” or within 3 h of medication
				Stretching	IN: Weight was increased as tolerated						“on” or within 3 h of medication
Paul et al. (2014)	IPD	40 (20/20)	NR	Power training	IN: 60% of the one repetition maximum.	Long period medium strength resistance exercise	2/12 wk	TUG			NA
				Usual care	Usual care						NA
Cugusi et al. (2015)	Hoehn-Yahr 1–3	20 (10/10)	67.4 ± 8	Nordic walking	IN: 60–80% HRR	Long period high intensity aerobic exercise	2/12 wk	UPDRS-III	6 MWT		Customary medication
				Usual care	Usual care						Customary medication
Fisher et al. (2008)	Hoehn-Yahr 1–2	30 (10/10/10)	62.3 ± 10.65	High-intensity group	IN: 3.0 METS and/or 75% of an AAMHR	Short period high intensity aerobic exercise	3/8 wk	UPDRS-III			Customary medication
				Low-intensity group	IN: 3.0 or fewer METS/50%HRR or less of their AAMHR for 45 min	Short period low intensity resistance exercise					Customary medication
				Zero-intensity group.	Usual care						Customary medication
Kurtais et al. (2008)	Hoehn-Yahr 1–3	24 (12/12)	64.8 ± 7.95	Training program on a treadmill	IN: 70–80% MHH either speed or inclination was gradually increased over time.	Short period high intensity aerobic exercise	3/6 wk	TUG			“on” state of medication
				No intervention	Usual care						Customary medication
Qutubuddin et al. (2013)	UPDRS-III >30	23 (13/10)	NR	Cycling program	IN: 61–80% of the individual's aerobic maximum.Weight was increased as tolerated	Short period high intensity aerobic exercise	2/8 wk	UPDRS-III	PDQ-39		“on” state (within 3 h)
				No intervention	Usual care						Customary medication
Schilling et al. (2010)	Hoehn-Yahr 1–2.5	15 (8/7)	59.2 ± 7.85	Hammer Strength	IN: Two sets for 8 repetitions, and the final set between 5 and 8 repetitions.	Short period high intensity resistance exercise	2/8 wk	6 MWT			“on” state of medication
				Standard care	Usual care						“on” state of medication
Dibble et al. (2015)	IPD	41(20/21)	68.4 ± 11.95	Resistance Exercise	IN: RPE was 13	Long period high intensity resistance exercise	12 wk	UPDRS-III	TUG	6 MWT	“on” medication state (1–1.5 h after medication intake).
				Standard care	Usual care						Customary medication
van der Kolk et al. (2018)	Hoehn-Yahr 1–2	37 (15/22)	NR	Aerobic exercise	IN: virtual reality software within their prescribed heart rate zone.	Long period low intensity aerobic exercise	3/6 M	UPDRS III	TWM	PDQ-39	OFF state
				No intervention	Usual care						OFF state
Carvalho et al. (2015)	Hoehn-Yahr 1–3	22 (5/8/9)	64.4 ± 11.7	Aerobic training	IN: 60% of the maximum VO_2max_ or 70% MHR	Long period high intensity aerobic exercise	2/12 wk	UPDRS III			NA
				Strength training	IN: 8–12 1RM	Long period high intensity resistance exercise					NA
				Physiotherapy	Usual care						NA

*N, number of participants; E, experimental group; C, control group; WK, week; M, month; NA, not applicable; IN, intensity; 1RM, of 1 repetition maximum; MHR, Maximal Heart Rate; VO_2max_, maximal oxygen consumption; UPDRS, Unified Parkinson's Disease Rating Scale; PDQ39, Parkinson's Disease Questionnaire 39; TUG, Timed Up and Go; TMW, 10-meter walk test; MDS-UPDRS, MDS-Unified Parkinson's Disease Rating Scale;6WMT, 6-min walk test*.

### Quality assessments of the selected literature

**Figure 2:**
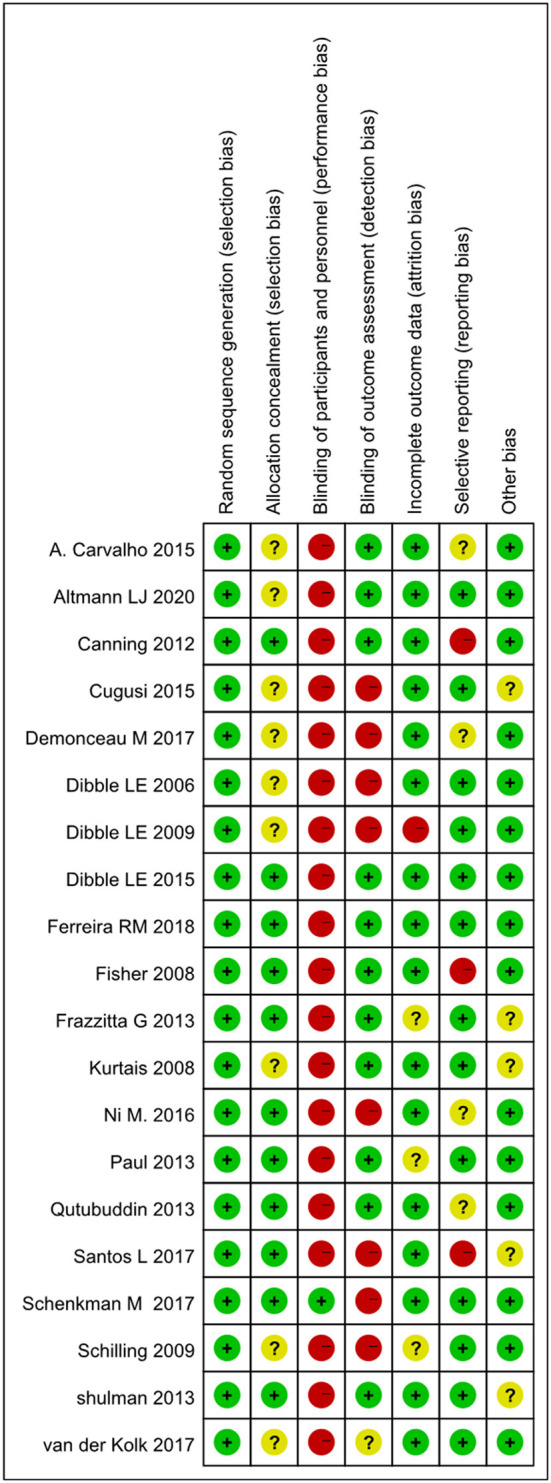
Combined percentage risk of bias in each risk domain for all included trials.

**Figure 3:**
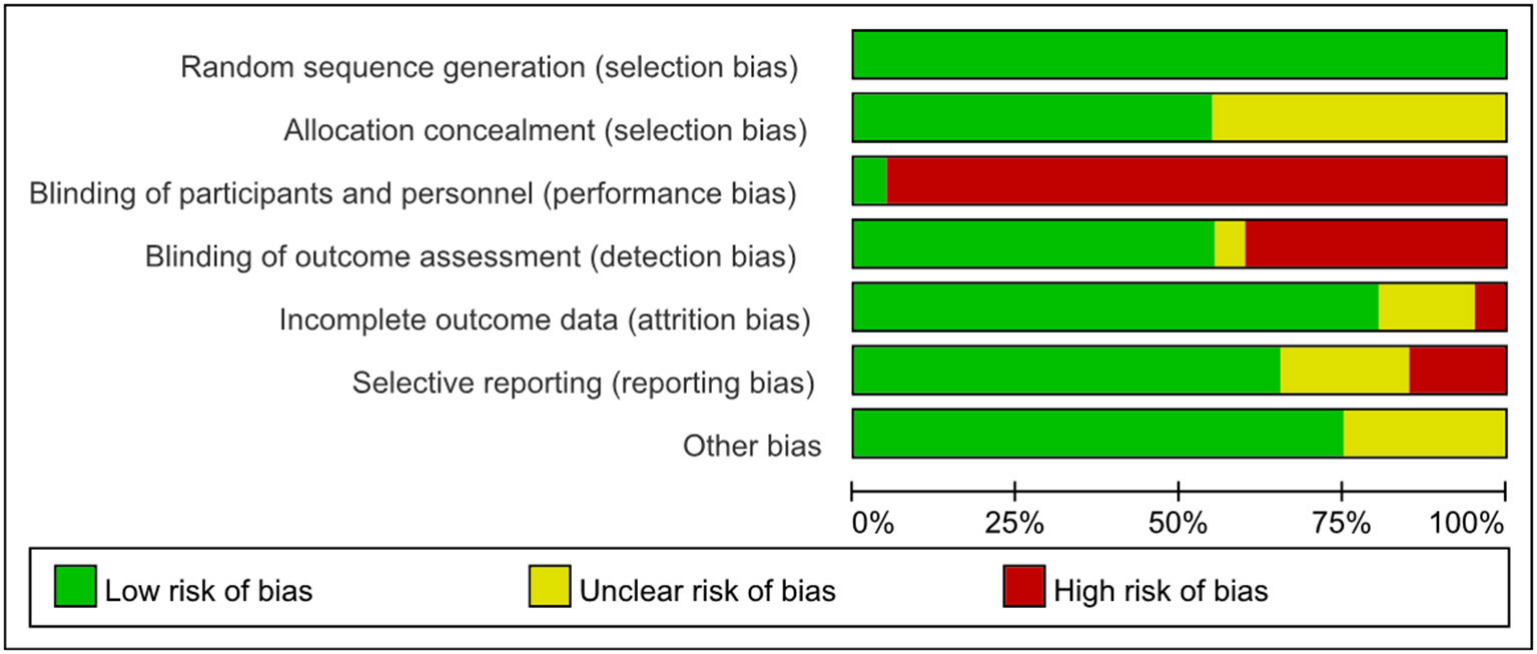
Risk of bias summaries for all exercise trials.

The individual and overall study level quality were presented in Figures 2, 3. All the 20 included trials reported adequate random sequence generation. Eleven RCTs described their approach of allocation concealment. Because the included articles are based on human research, it is difficult to apply blinding to the participants. One RCTs with low bias concerned blinding of performance bias, 11 RCTs with low bias with detection bias. Three RCTs with a high risk of allover bias originated from reporting bias and other bias, respectively.

### Results of direct meta-analysis

We performed a direct meta-analysis to investigate the efficacy of aerobic and resistance training of different intensity and period in PD. 14 direct studies were included in UPDRS III, 5 direct studies in TUG, 5 direct studies in 6MWT, 8 direct studies in TWM; and 5 direct studies in PDQ-39; as shown in Table 4. Based on strong heterogeneity (*p* > 0.05 or *I*^2^ ≤ 50%), fixed-effects models were selected to estimate the combined results of different comparisons for the following indicators: UPDRS III indicators: long period high intensity aerobic exercise vs. control, long period high intensity resistance training vs. control, short period high intensity aerobic exercise vs. control. TWM indicators: long period high intensity aerobic exercise vs. control, long period low intensity aerobic exercise vs. control; TUG indicator: long period high intensity aerobic exercise vs. control as TUG indicator. PDQ-39: long period high intensity aerobic exercise vs. control; but 6MWT: long period high intensity aerobic exercise vs. con comparison using random effects model. Thus, the results of the meta-analysis indicating that the differences between these long period high intensity aerobic exercise and long period low intensity aerobic exercise (95% CI <0) comparisons were significant and that different exercises had a therapeutic effect on PD, but none of the other differences between comparisons were statistically significant.

**Table 4:** Results of Direct meta-analysis.

**Variable**	**Group**	**k**	**SMD (95%CI)**	**Direct Evidence**	**I^2^ (%)**	**Model**
UPDRS III	LP-HI-AE VS. CON	4	−0.36 [−0.90; 0.17]	0.76	0	Fixed
	LP-HI-RT VS. CON	3	−0.20 [−0.79; 0.038]	0.87	0	Fixed
	LP-MI-AE VS. CON	1	−0.26 [−1.09; 0.57]	0.58		
	SP-HI-AE VS. CON	2	−0.31 [−1.10; 0.47]	0.8	0	Fixed
	SP-LI-RT VS. CON	1	0.21 [−0.91; 1.34]	0.6		
	LP-HI-AE VS. LP-HI-RT	1	−1.28 [−2.74; 0.18]	0.21		
	LP-HI-AE VS. LP-MI-AE	1	0.21 [−0.62; 1.04]	0.58		
	SP-HI-AE VS. SP-LI-RT	1	−0.22 [−1.35; 0.91]	0.6		
TUG	LP-HI-AE VS. CON	3	−0.39 [−0.82; 0.04]	0.85	0	Fixed
	LP-HI-RT VS. CON	1	−0.00 [−0.72; 0.72]	0.58		
	LP-HI-AE VS. LP-HI-RT	1	−0.44 [−1.16; 0.27]	0.58		
6MWT	LP-HI-AE VS. CON	3	0.21 [−0.68; 1.10]	0.84	0.81	Random
	LP-LI-AE VS. CON	1	1.06 [−0.40; 2.51]	0.59		
	LP-HI-AE VS. LP-LI-AE	1	−1.71 [−3.19;−0.23]	0.57		
TWM	LP-HI-AE VS. CON	3	−0.20 [−0.64; 0.34]	0.69	0	Fixed
	LP-HI-RT VS. CON	1	0.04 [−1.05; 1.14]	0.54		
	LP-LI-AE VS. CON	2	0.17 [−0.27; 0.61]	0.73	0	Fixed
	LP-HI-AE VS. LP-HI-RT	1	0.05 [−1.06; 1.17]	0.52		
	LP-HI-AE VS. LP-LI-AE	1	−0.20 [−0.78; 0.39]	0.52		
PDQ-39	LP-HI-AE VS. CON	3	0.18 [−0.28; 0.64]	0.83	0.05	Fixed
	LP-HI-RT VS. CON	1	−0.00 [−0.72; 0.72]	0.59		
	LP-HI-AE VS. LP-HI-RT	1	0.66 [−0.77; 1.38]	0.58		

*K, Number of studies; LP-HI-AE, long period high intensity aerobic exercise; LP-HI-RT, long period high intensity resistance training; LP-MI-AE, long period moderate intensity aerobic exercise; SP-HI-AE, short period high intensity aerobic exercise; SP-LI-RT, short period low intensity resistance training; LP-HI-AE, long period high intensity aerobic exercise; LP-HI-RT, long period high intensity resistance training; LP-LI-AE, long period low intensity aerobic exercise; UPDRSIII, Unified Parkinson's Disease Rating Scale; PDQ39, Parkinson's Disease Questionnaire 39; TUG, Timed Up and Go; TMW, 10-meter walk test; 6MWT, 6-min walk test*.

### Results of network meta-analysis

**Figure 4:**
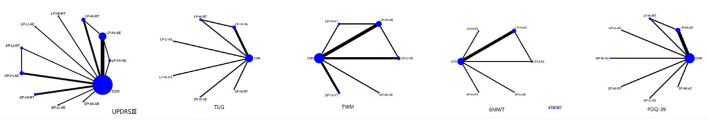
Network of evidence UPDRS-III outcome. SP-MI-AE, short period moderate intensity aerobic exercise; SP-LI-AE, short period low intensity aerobic exercise; SP-HI-RT, short period high intensity resistance training; SP-HI-AE, short period high intensity aerobic exercise; SP-LI-RT, short period low intensity resistance training; LP-LI-AE, long period low intensity aerobic exercise; LP-HI-RT, long period high intensity resistance training; LP-HI-AE, long period high intensity aerobic exercise; LP-MI-AE, long period moderate intensity aerobic exercise; CON, control group.

**Figure 5:**
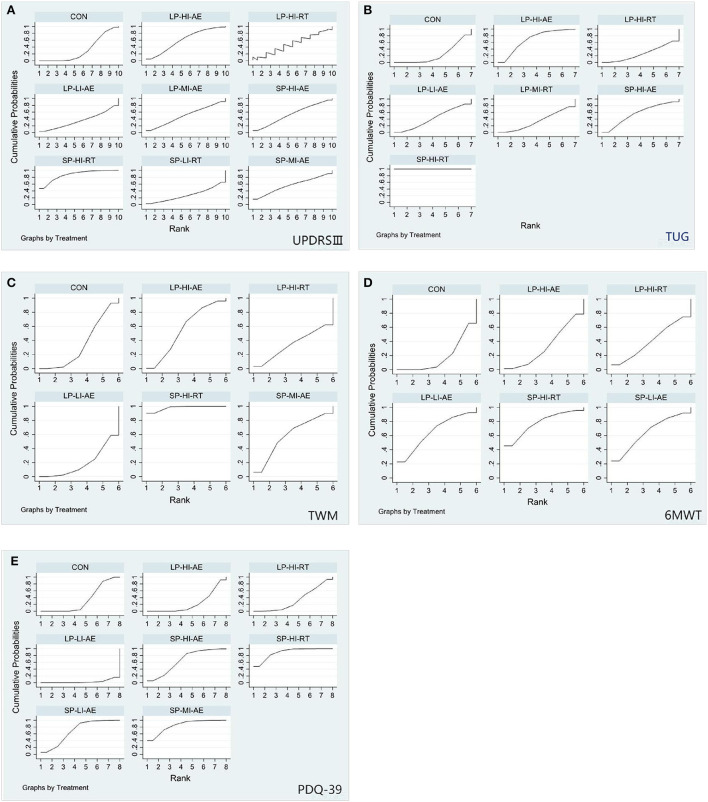
The rank probability of various interventions based on the SUCRA [**(A)** UPDRS-III outcome; **(B)** TUG outcome; **(C)** TWM outcome; **(D)** 6MWT outcome; **(E)** PDQ-39 outcome; SP-LI-AE, short period low intensity aerobic exercise; SP-HI-RT, short period high intensity resistance training; SP-HI-AE, short period high intensity aerobic exercise; SP-LI-RT, short period low intensity resistance training; LP-LI-AE, long period low intensity aerobic exercise; LP-HI-RT, long period high intensity resistance training; LP-HI-AE, long period high intensity aerobic exercise; LP-MI-AE, long period moderate intensity aerobic exercise; CON, control group].

**Figure 6:**
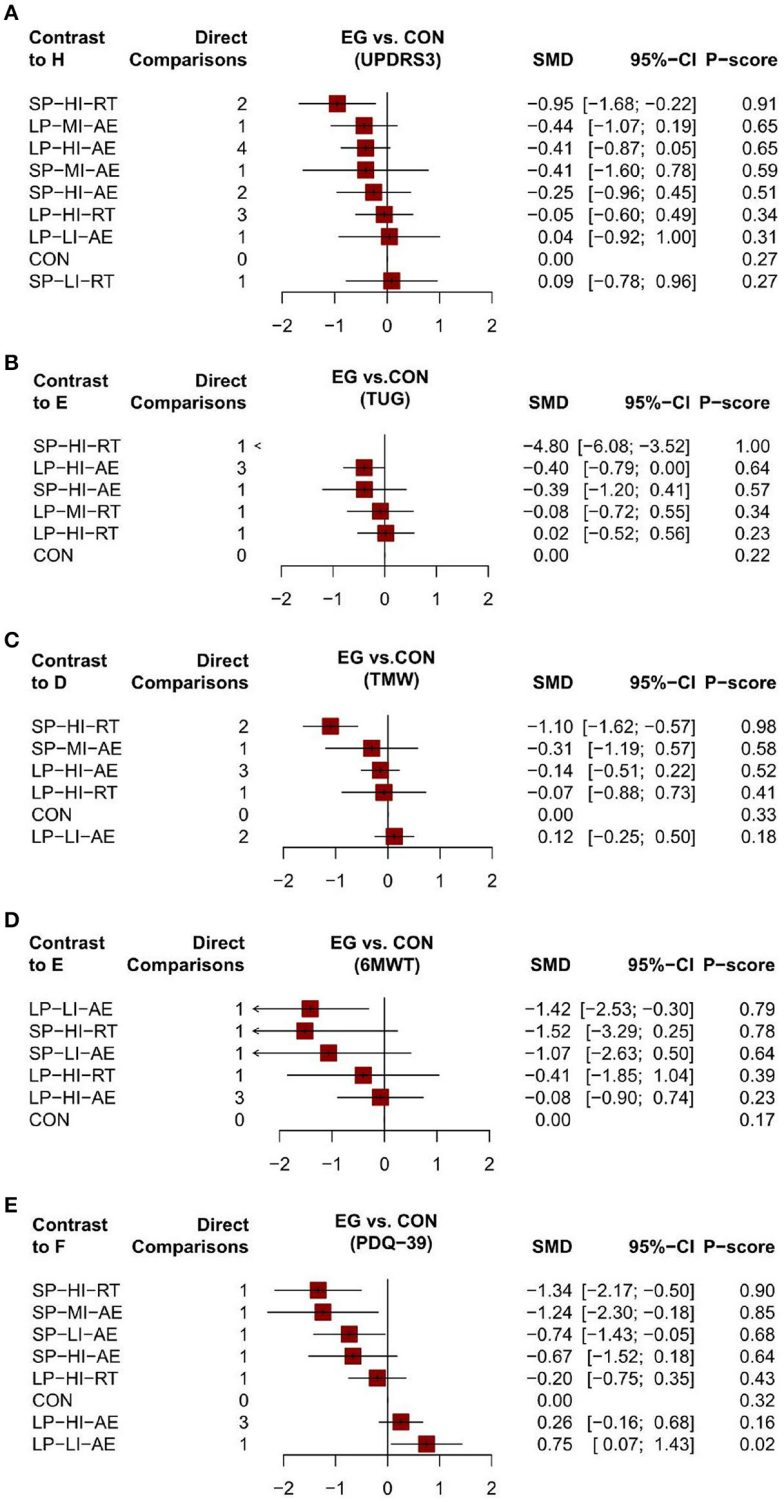
The forest plot of aerobic and resistance exercise outcomes [**(A)** UPDRS-III outcome; **(B)** TUG outcome; **(C)** TWM outcome; **(D)** 6MWT outcome; **(E)** PDQ-39 outcome; SP-MI-AE, short period moderate intensity aerobic exercise; SP-LI-AE, short period low intensity aerobic exercise; SP-HI-RT, short period high intensity resistance training; SP-HI-AE, short period high intensity aerobic exercise; SP-LI-RT, short period low intensity resistance training; LP-LI-AE, long period low intensity aerobic exercise; LP-HI-RT, long pe-riod high intensity resistance training; LP-HI-AE, long period high intensity aerobic exercise; LP-MI-AE, long period moderate intensity aerobic exercise; CON, control group; EG, experimental group; SMD, Mean Difference; 95%CI, confidence interval].

**Figure 7:**
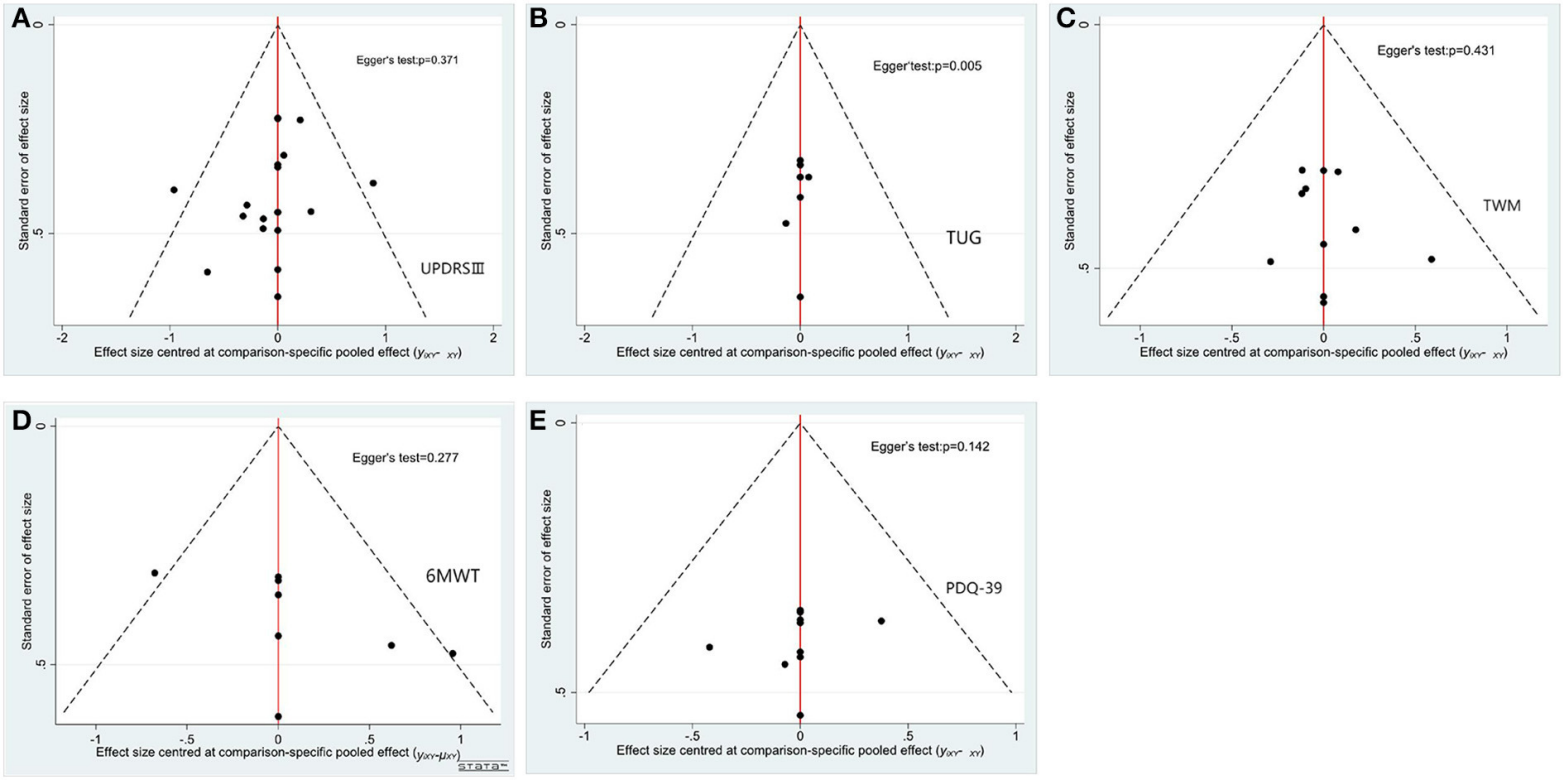
Funnel plots and bias of aerobic and resistance exercise outcomes [**(A)** UPDRS-III outcome; **(B)** TUG outcome; **(C)** TWM outcome; **(D)** 6MWT outcome; **(E)** PDQ-39 outcome; SP-MI-AE, short period moderate intensity aerobic exercise; SP-LI-AE, short period low intensity aerobic exercise; SP-HI-RT, short period high intensity resistance training; SP-HI-AE, short period high intensity aerobic exercise; SP-LI-RT, short period low intensity resistance training; LP-LI-AE, long period low intensity aerobic exercise; LP-HI-RT, long period high intensity resistance training; LP-HI-AE, long period high intensity aerobic exercise; LP-MI-AE, long period moderate intensity aerobic exercise].

Unified Parkinson's Disease Rating Scale Motor (UPDRS III). Network meta-analysis (12/20) studies (Dibble et al., 2006, 2015; Fisher et al., 2008; Canning et al., 2012; Qutubuddin et al., 2013; Frazzitta et al., 2014; Carvalho et al., 2015; Cugusi et al., 2015; Ferreira et al., 2018; Schenkman et al., 2018; van der Kolk et al., 2018; Vieira de Moraes Filho et al., 2020) involving 574 subjects with data provided between 11 different treatment nodes assessed motor function using UPDRS III measurements (Figure 4). The NMA of UPDRS III showed that short period high intensity resistance movement (SMD = −0.95; 95% CI −1.68 to −0.22) was significantly reduced compared with the control group. The pairwise meta-analysis showed that in comparison with the control group, short period high intensity resistance movement significantly decreased the UPDRS III score (Table 5A). Other comparisons were found to be statistically insignificant. The ranking of treatments based on cumulative probability plots and SUCRAs reveals that all exercises showed better results than the control group, except for short periods of low intensity resistance exercise. Short period high intensity resistance movement (SUCRA = 87%) was the most effective treatment and short period low intensity resistance exercise (27%) the least (Figures 5A, 6A). No evidence of publication bias was presented in Figure 7A. Quantification of the inconsistencies between direct and indirect comparisons using node-splitting methods and the design-by-treatment interaction model showed that all *p*-values exceeded 0.05 (*p*-value = 0.58), which indicated satisfactory consistency.

**Table 5A:** Network meta-analysis of the efficacy of UPDRSIII.

**SP-HI-RT**	NA	NA	NA	NA	NA	NA	−0.95 (−1.68; −0.22)	NA
−0.51 (−1.48; 0.45)	**LP-MI-AE**	−0.21 (−1.04; 0.62)	NA	NA	NA	NA	−0.26 (−1.09; 0.57)	NA
−0.54 (−1.41; 0.32)	−0.03 (−0.66; 0.60)	**LP-HI-AE**	NA	NA	−1.28 (−2.74; 0.18)	NA	−0.36 (−0.90; 0.17)	NA
−0.54 (−1.94; 0.86)	−0.03 (−1.38; 1.32)	−0.00 (−1.28; 1.28)	**SP-MI-AE**	NA	NA	NA	−0.41 (−1.60; 0.78)	NA
−0.70 (−1.71; 0.32)	−0.18 (−1.13; 0.76)	−0.16 (−1.00; 0.69)	−0.16 (−1.54; 1.23)	**SP-HI-AE**	NA	NA	−0.31 (−1.10; 0.47)	−0.22 (−1.35; 0.91)
−0.90 (−1.81; 0.01)	−0.39 (−1.20; 0.43)	−0.36 (−1.03; 0.32)	−0.36 (−1.67; 0.96)	−0.20 (−1.09; 0.69)	**LP-HI-RT**	NA	−0.20 (−0.79; 0.38)	NA
−0.99 (−2.20; 0.21)	−0.48 (−1.63; 0.67)	−0.45 (−1.52; 0.62)	−0.45 (−1.98; 1.08)	−0.30 (−1.49; 0.90)	−0.09 (−1.20; 1.01)	**LP-LI-AE**	0.04 (−0.92; 1.00)	NA
−0.95 (−1.68; −0.22)	−0.44 (−1.07; 0.19)	−0.41 (−0.87; 0.05)	−0.41 (−1.60; 0.78)	−0.25 (−0.96; 0.45)	−0.05 (−0.60; 0.49)	0.04 (−0.92; 1.00)	**CON**	−0.21 (−1.34; 0.91)
−1.04 (−2.18; 0.10)	−0.53 (−1.60; 0.55)	−0.50 (−1.49; 0.49)	−0.50 (−1.98; 0.98)	−0.34 (−1.21; 0.53)	−0.14 (−1.17; 0.89)	−0.05 (−1.34; 1.25)	−0.09 (−0.96; 0.78)	**SP-LI-RT**

Timed-Up-and-Go Test (TUG). We conducted a network meta-analysis of the TUG results, which showed that short period high intensity resistance movement (SMD = −4.80, 95% CI −6.08 to −3.52) and long period high intensity aerobic exercise (SMD = −0.40, 95% CI −0.79 to 0.00) were more conducive to lower TUG scores than controls Table 5B. We assessed the ranking of the various treatments according to TUG scores and found that short period high intensity resistance movement ranked highest (SUCRA = 92.6%) Figures 5B, 6B. All other treatments were better than the control group, but there was no significant difference. Quantification of inconsistency between direct and indirect comparisons using a node-splitting approach and a design-treatment interaction model showed that all p-values exceeded 0.05, which indicates satisfactory agreement. Figure 7B shows no evidence of publication bias.

**Table 5B:** Network meta-analysis of the efficacy of TUG.

**SP-HI-RT**	NA	NA	NA	NA	NA	−4.80 (−6.08; −3.52)
−4.29 (−5.65; −2.92)	**LP-HI-AE**	NA	NA	NA	−0.44 (−1.16; 0.27)	−0.54 (−1.11; 0.03)
−4.41 (−5.92; −2.90)	−0.12 (−1.07; 0.83)	**SP-HI-AE**	NA	NA	NA	−0.39 (−1.20; 0.41)
−4.61 (−6.05; −3.18)	−0.33 (−1.15; 0.50)	−0.21 (−1.25; 0.84)	**LP-HI-AE**	NA	NA	−0.19 (−0.85; 0.47)
−4.72 (−6.14; −3.29)	−0.43 (−1.24; 0.37)	−0.31 (−1.34; 0.72)	−0.10 (−1.02; 0.81)	**LP-MI-RT**	NA	−0.08 (−0.72; 0.55)
−4.76 (−6.16; −3.37)	−0.48 (−1.04; 0.08)	−0.36 (−1.34; 0.63)	−0.15 (−1.02; 0.71)	−0.05 (−0.90; 0.80)	**LP-HI-RT**	0.00 (−0.72; 0.72)
−4.80 (−6.08; −3.52)	−0.52 (−1.01; −0.02)	−0.39 (−1.20; 0.41)	−0.19 (−0.85; 0.47)	−0.08 (−0.72; 0.55)	−0.04 (−0.60; 0.52)	**CON**

10-meter walk test (TWM). The results of the network meta-analysis showed that short period high intensity resistance movement (SMD = −1.10; 95% CI −1.62 to −0.57) was greatly different from the control group Table 5C. The SUCRA (Figures 5C, 6C) showed that all the results were better than the control group except for the long period low intensity aerobic exercise treatment and short period high intensity resistance movement scored the highest in the TWM (SUCRA = 98 %). Figure 7C shows no evidence of publication bias. All other exercises were better than the control group, but there was no significant difference. All p-values exceeded 0.05, which indicates satisfactory agreement.

**Table 5C:** Network meta-analysis of the efficacy of TWM.

**SP-HI-RT**	NA	NA	NA	−1.10 (−1.62; −0.57)	NA
−0.79 (−1.82; 0.24)	**SP-MI-AE**	NA	NA	−0.31 (−1.19; 0.57)	NA
−0.95 (−1.59; −0.31)	−0.16 (−1.12; 0.79)	**LP-HI-AE**	0.05 (−1.06; 1.17)	−0.20 (−0.64; 0.24)	−0.20 (−0.78; 0.39)
−1.02 (−1.98; −0.07)	−0.23 (−1.43; 0.96)	−0.07 (−0.87; 0.73)	**LP-HI-RT**	0.04 (−1.05; 1.14)	NA
−1.10 (−1.62; −0.57)	−0.31 (−1.19; 0.57)	−0.14 (−0.51; 0.22)	−0.07 (−0.88; 0.73)	**CON**	−0.17 (−0.61; 0.27)
−1.22 (−1.87; −0.58)	−0.43 (−1.39; 0.53)	−0.27 (−0.69; 0.15)	−0.20 (−1.06; 0.66)	−0.12 (−0.50; 0.25)	**LP-LI-AE**

6 Minute Walk Test (6MWT). Paired mesh meta-analysis showed a significant difference between short periods high intensity resistance exercise (SMD = 1.06; 95% CI −0.63 to 2.75) and control group Table 5D. There was a significant difference in the long period low intensity of aerobic exercise (SMD = −0.58; 95% CI 0.28 to 2.55) compared to the control group (Figures 5D, 6D). all other exercises were better than the control group, but there was no significant difference. No evidence of publication bias Figure 7D.

**Table 5D:** Network meta-analysis of the efficacy of 6MWT.

**SP-HI-RT**	−0.46 (−3.06, 2.13)	−0.45 (−3.11, 2.20)	−1.11 (−3.70, 1.47)	−1.29 (−3.51, 0.93)	−1.06 (−2.75, 0.63)
0.46 (−2.13, 3.06)	**LP-LI-AE**	0.01 (−2.45, 2.47)	−0.65 (−3.03, 1.73)	−0.83 (−2.80, 1.15)	−0.88 (−3.49, 1.73)
0.45 (−2.20, 3.11)	−0.01 (−2.47, 2.45)	**SP-LI-AE**	−0.66 (−3.11, 1.79)	−0.84 (−2.90, 1.22)	−1.52 (−3.49, 0.45)
1.11 (−1.47, 3.70)	0.65 (−1.73, 3.03)	0.66 (−1.79, 3.11)	**LP-HI-RT**	−0.18 (−2.15, 1.79)	−0.41 (−2.09, 1.28)
1.29 (−0.93, 3.51)	0.83 (−1.15, 2.80)	0.84 (−1.22, 2.90)	0.18 (−1.79, 2.15)	**LP-HI-AE**	−0.23 (−1.25, 0.79)
1.06 (−0.63,−2.75)	0.88 (−1.73, 3.49)	1.52 (−0.45, 3.49)	0.41 (−1.28, 2.09)	0.23 (−0.79, 1.25)	**CON**

The 39-item Parkinson's Disease Questionnaire (PDQ-39). The NMA for the PDQ-39 showed that compared to the control group, there were significant differences in short period high intensity resistance exercise (SMD = −1.34; 95% CI −2.17 to 0.50), short periods of moderate-intensity aerobic training (SMD = −1.24; 95% CI −2.3 to −0.18); and short periods of low-intensity aerobic training (SMD = −0.74; 95% CI −1.43 to −0.05) Table 5E. The ranking of treatments based on cumulative probability plots and SUCRA (Figures 5E, 6E) showed that the most effective treatment was short period high intensity resistance movement exercise (SUCRA = 90%). Figure 7E shows no evidence of publication bias.

**Table 5E:** Network meta-analysis of the efficacy of PDQ-39.

**SP-HI-RT**	NA	NA	NA	NA	−1.34 (−2.17; −0.50)	NA	NA
−0.10 (−1.45; 1.25)	**SP-MI-AE**	NA	NA	NA	−1.24 (−2.30; −0.18)	NA	NA
−0.60 (−1.68; 0.48)	−0.50 (−1.77; 0.76)	**SP-LI-AE**	NA	NA	−0.74 (−1.43; −0.05)	NA	NA
−0.67 (−1.86; 0.52)	−0.57 (−1.93; 0.79)	−0.07 (−1.17; 1.02)	**SP-HI-AE**	NA	−0.67 (−1.52; 0.18)	NA	NA
−1.14 (−2.14; −0.14)	−1.04 (−2.24; 0.15)	−0.54 (−1.42; 0.34)	−0.47 (−1.48; 0.54)	**LP-HI-RT**	0.00 (−0.72; 0.72)	−0.66 (−1.38; 0.07)	NA
−1.34 (−2.17; −0.50)	−1.24 (−2.30; −0.18)	−0.74 (−1.43; −0.05)	−0.67 (−1.52; 0.18)	−0.20 (−0.75; 0.35)	**CON**	−0.18 (−0.64; 0.28)	−0.75 (−1.43; −0.07)
−1.59 (−2.53; −0.66)	−1.50 (−2.64; −0.36)	−1.00 (−1.80; −0.19)	−0.93 (−1.87; 0.02)	−0.45 (−1.01; 0.10)	−0.26 (−0.68; 0.16)	**LP-HI-AE**	NA
−2.09 (−3.16; −1.01)	−1.99 (−3.25; −0.73)	−1.49 (−2.46; −0.52)	−1.42 (−2.51; −0.33)	−0.95 (−1.82; −0.07)	−0.75 (−1.43; −0.07)	−0.49 (−1.29; 0.31)	**LP-LI-AE**

*Relative effect sizes of efficacy at post-treatment according to network meta-analysis. Treatments are orders in the rank of their chance of being the best treatment. Numbers in bule boxes are SUCRA (the surface under the cumulative ranking curve) values and their CrIs (credible intervals). Significant pairwise comparisons are highlighted in red. For efficacy in post-treatment, standardized mean differences (SMDs) less than 0 favor the column-defining treatment. UPDRS, Unified Parkinson's Disease Rating Scale; PDQ39, Parkinson's Disease Questionnaire 39; TUG, Timed Up and Go; TMW, 10-meter walk test; MDS-UPDRS, MDS-Unified Parkinson's Disease Rating Scale; 6WMT, 6-min walk test; LP-HI-AE, long period high intensity aerobic exercise; LP-HI-RT, long period high intensity resistance training; LP-MI-AE, long period moderate intensity aerobic exercise; SP-HI-AE, short period high intensity aerobic exercise; SP-LI-RT, short period low intensity resistance training; LP-HI-AE, long period high intensity aerobic exercise; LP-HI-RT, long period high intensity resistance training; LP-LI-AE, long period low intensity aerobic exercise*.

## Discussion

The aim of this study was to perform a network meta-analysis of aerobic and resistance exercise therapy in patients with PD. To our knowledge, to date, no other review has performed a network meta-analysis of aerobic and resistance training with different outcomes on motor function in PD, as well as no further classification of exercise doses for aerobic and resistance training. Our results show that different aerobic and resistance training showed effects in PD patients, reflecting the complementary efficacy of aerobic and resistance training in the non-pharmacological treatment of PD, as well as the prominent role of high intensity training based on different doses of exercise modalities. In difference to the previous network meta-analysis of multiple exercise modalities by Tang et al. (2019), the focus of this paper is that since aerobic and resistance are the most common training modalities, studies of specific intensity cycles can provide a broader range of dose recommendations for future studies, rather than being limited to one specific study.

In particular, the studies in this paper show the effectiveness of short periods high intensity exercise, and many studies in recent years have highlighted the value of high intensity resistance training, with previous studies confirming muscle atrophy, weakness, low muscle strength, and fatigability associated with aging in PD, and demonstrating the role of resistance exercise training at high intensity in PD patients (Leek et al., 2001; Bickel et al., 2011; Chalé et al., 2013). Kelly et al. (2018) concluded that PD patients perform exercise training at sufficient intensity to achieve robust adaptation of skeletal muscle, that preferential hypertrophy of type II muscle fibers is a hallmark adaptation of resistance training, and that resistance training can counteract aging type II atrophy by promoting regeneration.

In comparison with controls, from pooled analysis, short period high intensity resistance movement (SMD = −0.95; 95% CI −1.68 to −0.22) was much more effective than other aerobic and resistance training in reducing UPDRS-III motor symptoms. Our results complement previous studies (Egger et al., 2003), expand on existing treatments, and are consistent with the recommendations in the ACSM (Riebe et al., 2015) for providing regular moderate to high intensity resistance training advice for PD. By contrast, the score of short period low intensity resistance movement (SMD = 0.09; 95% CI 0.78 to 0.96) lags behind in UPDRS III scores. Appropriate intensity has been identified as a key determinant of neuromuscular adaptations to strength training, since the exercise needs to reach a certain intensity to achieve neuromuscular changes, and high intensity leads to greater neuromuscular adaptations (Petzinger et al., 2013; Sallis et al., 2015). Furthermore, several studies have confirmed that potential mechanisms for high intensity resistance training induced neuromuscular remodeling and improved motor function may be related to the up regulation of genes that enhance muscle development, and that central motor path-ways still exhibit altered neuroplasticity following resistance training despite the advanced age and neurological dysfunction of PD (Lötzke et al., 2015; Saltychev et al., 2016). At the same time, we observed that other intensities and periods of both aerobic and resistance training showed advantages over the control group, which further confirms the improvement of aerobic and resistance training on motor aspects in PD. Similar results were obtained with resistance training conducted by Corcos et al. (2013), who carried out progressive resistance training in PD for two years and showed statistically and clinically significant reductions in UPDRS-III scores, suggesting resistance training as a useful adjunct treatment to improve motor signs in PD. Interestingly, animal studies in PD models have also shown that locomotor training can reduce α-synuclein aggregation and improve both motor and cognitive function (Zhou et al., 2017) and exercise increases neuronal activation and dopamine increases in the basal ganglia (Lau et al., 2011), which may have an impact on UPDRS scores, and regular exercise therapy may reverse or attenuate the underlying neurodegenerative process in PD, ultimately leading to improved UPDRS scores.

As common indicators of motor performance and gait, the results of TUG, 6MWT, and TWM suggest that short period low intensity resistance movement can be used as an adjunct to improve motor symptoms and prevent falls in Parkinson's patients. This is consistent with the results of a previous meta-analysis (Santos et al., 2017; Tang et al., 2019). A study by Santos et al. (2017) found that resistance training improved freezing gait in PD patients. The strength improvement brought by resistance training may facilitate the activation of balance related muscle groups. This mechanism may be related to the fact that high intensity resistance training improves cardiovascular conditioning and increases the level of skeletal muscle force production (Dibble et al., 2015). In addition, high intensity resistance training may help to enhance neural drive (Dibble et al., 2009b), leading to better postural control and thus improved gait. Also, long period high intensity aerobic exercise (SMD = 0.40; 95%CI 0.78 to 0.96) showed significant differences from controls in TUG, which is consistent with the conclusion of a previous meta-analysis that both aerobic and resistance training were effective in improving patients' gait (Tomlinson et al., 2012). Previous studies emphasizing high intensity aerobic exercise show promise for improving PD symptoms (Alberts et al., 2011; Schenkman et al., 2018). For example, Fisher et al. (2008) used treadmill training to have patients with Parkinson's disease perform gait training at a faster pace than they would have chosen for themselves, and over an 8-week period, the patients improved gait and balance parameters as the training was progressively improved, while showing a decrease in cortical motor excitability *via* transcranial magnetic stimulation.

For the quality of life, the PDQ-39 results show that short cycles of high intensity resistance exercise improve the quality of life in PD patients with mild or moderate symptoms. This is consistent with the previous observation (Dibble et al., 2009a) in which eccentric training was found to have significantly improved patients' quality of life and after resistance training patients experienced some positive changes in habitual behaviors that could be attributed to the training. Participants in this research enjoyed an improved quality of life who felt less fatigued, had a better appetite, and slept and rested well. In conclusion, we recommend that people with Parkinson's disease incorporate resistance movement into their physical activity routine to improve motor symptoms and enhance their quality of life (Wu et al., 2017). However, the above benefits may arise from the design of the research, the sample size and the measurement problems of the research, or the fidelity of implementation in other issues.

## Advantages

To our knowledge, this network meta-analysis is the first study to compare the effects of aerobic and resistance training on Parkinson's patients and to further divide them according to exercise periods and intensity when determining the beneficial guidance of different exercise doses for PD. It also explores the ranking of various PD treatments based on a comprehensive ranking that identifies the best option for improving movement and quality of life for Parkinson's patients. A variety of physiotherapy methods are applied to treat people with Parkinson's disease, but previous reviews have focused on simply one type of physiotherapy (Shu et al., 2014; Lamotte et al., 2015).

## Limitations

Following are the limitations of our study. In terms of exercise outcomes, the number of studies is small and direct comparisons of exercise assessments are lacking the studies included in our NMA used the results as continuous variables as a basis. At the same time, the more restrictive classification of exercise intensity and exercise period resulted in many mixed-mode exercises not being included, which may have led to incomplete results. Future studies need to further refine the way exercise doses are studied. The classification of motion periods is a gap in current research, and despite our efforts to try to find more objective criteria, unfortunately there are no relevant studies, which is the limitation of this paper and requires further investigation in the future. In the analysis section, we extracted the mean, SD, and sample size values at the last observation for analysis. However, some studies lost the above data, which made the number of available studies even smaller. Also, the participant was blinding at the time of inclusion in some of the literature, and it was difficult to ensure participant blinding in exercise therapy, which may lead to bias. The quality of several studies potentially threatened the validity of our study. Future studies should explore a wider range of metrics for evaluating different types of exercise doses, include more types of studies, and increase the credibility of the studies. Therefore, based on the complexity of the Network meta, the results of this study should be interpreted with caution due to the small number of studies.

## Conclusion

In conclusion, our network meta-analysis showed that short period high intensity resistance training, as a complement to pharmacotherapy, improved motor symptoms in PD better compared to aerobic and resistance exercise of other period and intensity. However, both aerobic and resistance training of different intensities and cycles showed some effects, and aerobic and resistance training can be recommended as types of exercise for PD. In the future, we need more high quality multicentre randomized controlled trials to confirm our findings.

## Data availability statement

The original contributions presented in the study are included in the article/Supplementary material, further inquiries can be directed to the corresponding author.

## Author contributions

XZ served as principal author and had full access to all the data in the study, takes responsibility for the accuracy of the data analysis, and the integrity of the data. RW and JW contributed to the conception and design. XZ, RW, and JW contributed to data acquisition and interpretation. XZ and JW contributed to draft of the manuscript. XZ, JW, and XG contributed to revise of the article and final approval. All authors contributed to the article and approved the submitted version.

## Funding

This research was supported by the Research and Application of Functional Assessment and Diagnosis, Rehabilitation Physical Training, and Injury Risk Prevention Integrated Protection System for Chinese Excellent Weightlifters, basic 15–33.

## Conflict of interest

The authors declare that the research was conducted in the absence of any commercial or financial relationships that could be construed as a potential conflict of interest.

## Publisher's note

All claims expressed in this article are solely those of the authors and do not necessarily represent those of their affiliated organizations, or those of the publisher, the editors and the reviewers. Any product that may be evaluated in this article, or claim that may be made by its manufacturer, is not guaranteed or endorsed by the publisher.
