# Supplementary material for: Effectiveness of aerobic and resistance training on the motor symptoms in Parkinson's disease: Systematic review and network meta-analysis
Source: Front Aging Neurosci. 2022 Aug 1;14:935176. doi: 10.3389/fnagi.2022.935176 (PMC9376630; doi:10.3389/fnagi.2022.935176)
Supplement: Supplementary file 1 [file Data_Sheet_1.pdf]

Supplementary Material

1 Supplementary Data

1.1 Supplementary 1. Search strategy for each database

Database: PubMed <inception to April 1 2022>

#19	Search: ((Parkinson disease[MeSH Terms]) AND ((((exercise*[MeSH Terms]) OR (resistance training[MeSH Terms])) OR (aerobic exercise[MeSH Terms])) OR ("aerobic exercise" or "body weight support treadmill" or "gait training" or "high-speed resistance training" or "multicomponent exercise program" or "multidisciplinary exercise program" or "Nordic Walking" or Physiotherapy or pilates or "power training"or "treadmill training" or "walking"))) AND (((((((randomized controlled trial[Publication Type]) OR (controlled clinical trial[Publication Type])) OR (randomized[Title/Abstract])) OR (placebo[Title/Abstract])) OR (trial[Title])) OR (clinical trials as topic[MeSH Terms])) NOT ((animals[MeSH Terms]) NOT (humans[MeSH Terms])))	957
#18	Search: ((((((randomized controlled trial[Publication Type]) OR (controlled clinical trial[Publication Type])) OR (randomized[Title/Abstract])) OR (placebo[Title/Abstract])) OR (trial[Title])) OR (clinical trials as topic[MeSH Terms])) NOT ((animals[MeSH Terms]) NOT (humans[MeSH Terms]))	1,260,928
#17	Search: (((((randomized controlled trial[Publication Type]) OR (controlled clinical trial[Publication Type])) OR (randomized[Title/Abstract])) OR (placebo[Title/Abstract])) OR (trial[Title])) OR (clinical trials as topic[MeSH Terms])	1,309, 148
#16	Search: (((exercise*[MeSH Terms]) OR (resistance training[MeSH Terms])) OR (aerobic exercise[MeSH Terms])) OR ("aerobic exercise" or "body weight support treadmill" or "gait training" or "high-speed resistance training" or "multicomponent exercise program" or "multidisciplinary exercise program" or "Nordic Walking" or Physiotherapy or pilates or "power training"or "treadmill training" or "walking")	511, 184

#15	Search: (animals[MeSH Terms]) NOT (humans[MeSH Terms])	4,988,807
#14	Search: humans[MeSH Terms]	20,350,308
#13	Search: animals[MeSH Terms]	25,339, 115
#12	Search: clinical trials as topic[MeSH Terms]	372,490
#11	Search: trial[Title]	259,908
#10	Search: randomly[Title/Abstract]	380,597
#9	Search: placebo[Title/Abstract]	233,628

#8	Search: randomized[Title/Abstract]	606,343
#7	Search: controlled clinical trial[Publication Type]	655,490
#6	Search: randomized controlled trial[Publication Type]	565,571
#5	Search: "aerobic exercise" or "body weight support treadmill" or "gait training" or "high-speed resistance training" or "multicomponent exercise program" or "multidisciplinary exercise program" or "Nordic Walking" or Physiotherapy or pilates or "power training"or "treadmill training" or "walking"	307,232
#4	Search: aerobic exercise[MeSH Terms]	229, 133
#3	Search: resistance training[MeSH Terms]	11,046
#2	Search: exercise*[MeSH Terms]	323,846
#1	Search: Parkinson disease[MeSH Terms]	75,926

Database: OvidMEDLINE(R) <1946 to April 1 2022>

Search Strategy: --

1 Parkinson$.mp. (127808)

2 exp parkinson disease/ (75660)

3 (aerobic exercise or body weight support treadmill or exercise$ or high-speed resistance training or multicomponent exercise program or multidisciplinary exercise program or power training or treadmill training or walking).mp. (446305)

4 exp resistance training/ (11007)

5 exp exercise$/(228521)

6 randomized controlled trial.pt. (562738)

7 controlled clinical trial.pt. (94123)

8 randomized.ab. (488562)

9 clinical trials as topic.sh. (199625)

10 randomly.ab. (323892)

11 trial.ti. (228179)

12 exp clinical trial/ (933680)

13 exp randomized controlled trials/ (157760)

14 exp cross-over studies/ (53141) 15
(clinic$ adj2 trial).mp. (748029)

16 (random$ adj5 control$ adj5 trial$).mp. (782409)

17 (crossover or cross-over).mp. (91502)

18 randomi$.mp. (914837)

19 (random$ adj5 (assign$ or allocat$ or assort$ or reciev$)).mp. (247521) 20 1
or 2 (127808)

21 3 or 4 or 5(479663)

22 6 or 7 or 8 or 9 or 10 or 11 or 12 or 13 or 14 or 15 or 16 or 17 or 18 or

19(1704442)

23 20 and 21 and 22 (1072)

Database: Embase <1974 to 2022 April 2>

Search Strategy:

----

1 Parkinson$.mp. (233,509)

2 exp parkinson disease/ (178, 129)

3 (aerobic exercise or body weight support treadmill exercise$ or Exercise Movement Techniques or high-speed resistance training or hydrotherapy or multicomponent exercise program or multidisciplinary exercise program or power training or resistance training or stretch or treadmill training).mp. (446555)

4 exp resistance training/ (23333)

5 exp exercise$/ (400792)

6 exp aerobic exercise/ (19473)

7 exp treadmill exercise/ (20062)

8 exp power training/ (65)

13 randomized.ab. (806723)

14 randomly.ab. (502808)

15 trial.ti. (362098)

16 exp clinical trial/ (1704827)

17 exp randomized controlled trials/ (707892)

18 exp cross-over studies/ (69980) 19
(clinic$ adj2 trial).mp. (1639410)

20 (random$ adj5 control$ adj5 trial$).mp. (961796)

21 (crossover or cross-over).mp. (123532)

22 randomi$.mp. (1323771)

23 (random$ adj5 (assign$ or allocat$ or assort$ or reciev$)).mp. (209949) 24 1
or 2 (233613)

25 3 or 4 or 5 or 6 or 7 or 8(817092)

26 13 or 14 or 15 or 16 or 17 or 18 or 19 or 20 or 21 or 22 or 23 (2920073) 27
24 and 25 and 26 (754)

Database: PsycINFO <1806 to 2022 April 2>

Search Strategy:

Set	No. Searched for Databases Results		
S1	Parkinson*	APA PsycInfo®	39453
S2	mainsubject(parkinson disease)	APA PsycInfo®	25842
S3	su((aerobic exercise or body weight support treadmill exercise$ or Exercise Movement Techniques or high-speed resistance training or hydrotherapy or multicomponent exercise program or multidisciplinary exercise program or power training or resistance training or stretch or treadmill training))	APA PsycInfo®	63328
S4	su(exercise$)	APA PsycInfo®	39377
S6	su(physical activity)	APA PsycInfo®	39490
S7	ab(randomized)	APA PsycInfo®	83500
S8	ab(randomly)	APA PsycInfo®	75844
S9	ti(trial)	APA PsycInfo®	41193
S10	ab(clinical trial)	APA PsycInfo®	51602
S11	ab(randomized controlled trials)	APA PsycInfo®	36991
S12	ab(cross-over studies)	APA PsycInfo®	2076
S13	ab(crossover studies)	APA PsycInfo®	5363
S14	ab(randomi*)	APA PsycInfo®	83917
S15	su(animals)	APA PsycInfo®	459210
S16	S1 OR S2	APA PsycInfo®<br><br>These databases are searched for part ofyour query.	39453
		APA PsycInfo®	

S17	S3 OR S4 OR "S5"	These databases are searched for part ofyour query.	63473
S18	S6 OR S7 OR "S8" OR "S9" OR "S10" OR "S11" OR "S12" OR "S13" OR "S14"	APA PsycInfo®<br>These databases are searched for part ofyour query.	120428
S19	S16 AND S17	APA PsycInfo®<br>These databases are searched for part ofyour query.	1074
S20	S18 AND S19	APA PsycInfo®<br>These databases are searched for part ofyour query.	277
S21	S20 NOT S15	APA PsycInfo®<br>These databases are searched for part ofyour query.	251

Cochrane

#1 MeSH descriptor: [Parkinson disease] explode all trees (4648)

#2 (aerobic exercise or body weight support treadmill or high-speed resistance training or hydrotherapy or multicomponent exercise program or multidisciplinary exercise program or Nordic Walking or power training or resistance training or treadmill training or walking) in Trials (Word variations have been searched) (53228)

#3 MeSH descriptor: [resistance training] explode all trees (14740)

#4 MeSH descriptor: [exercise] explode all trees (111868)

#5 MeSH descriptor: [aerobic exercise] explode all trees (373)

#6 MeSH descriptor: [treadmill exercise] explode all trees (2215)

#7 MeSH descriptor: [power training] explode all trees (699)

#8 #2 or #3 or #4 or #5 or #6 or #7 (131956)

#9 #1 and #12 (852)

Database: Web ofScience <1965 to 2022 April 2>

7 **2,890** #6 AND #5 AND #1
Indexes=SCI-EXPANDED, SSCI, A&HCI, CPCI-S, CPCI-SSH, BKCI-S, BKCI-SSH, ESCI, CCR-EXPANDED, IC Timespan=Allyears

6 **570,164** #4 OR #3 OR #2
Indexes=SCI-EXPANDED, SSCI, A&HCI, CPCI-S, CPCI-SSH, BKCI-S, BKCI-SSH, ESCI, CCR-EXPANDED, IC Timespan=Allyears

5 **7,531,920** **TOPIC:** ((“randomized controlled trial*” or “controlled clinical trial” or “random*” or “clinical trial*” or randomly or trial or “clinical trial” or “randomized controlled trial*” or “cross-over studies” or clinic*))
Indexes=SCI-EXPANDED, SSCI, A&HCI, CPCI-S, CPCI-SSH, BKCI-S, BKCI-SSH, ESCI, CCR-EXPANDED, IC Timespan=Allyears

4 **501,011** **TOPIC:** (Exercise* or “Exercise Program, Weight-Bearing” or “Exercise Programs, Weight-Bearing” or “Weight Bearing Exercise Program” or “Weight-Bearing Exercise Programs” Exercise* or “Physical Activity” or “Activities, Physical” or “Activity, Physical” or “Physical Activities” or “Exercise, Physical” or “Exercises, Physical” or “Physical Exercise” or “Physical Exercises” or “Exercise, Isometric” or “Exercises, Isometric” or “Isometric Exercises” or “Isometric Exercise” or “Exercise, Aerobic” or “Aerobic Exercise” or “Aerobic Exercises” or “Exercises, Aerobic” or “Exercise Training” or “Exercise Trainings” or “Training, Exercise” or “Trainings, Exercise”)
Indexes=SCI-EXPANDED, SSCI, A&HCI, CPCI-S, CPCI-SSH, BKCI-S, BKCI-SSH, ESCI, CCR-EXPANDED, IC Timespan=Allyears

3 **19,016** **TOPIC:** ("Resistance training” or “Training, Resistance” or “Strength Training” or “Training, Strength” or “Weight-Lifting Strengthening Program” or “Strengthening Program, Weight-Lifting” or “Strengthening Programs, Weight-Lifting” or “Weight Lifting Strengthening Program” or “Weight-Lifting Strengthening Programs” or “Weight-Lifting Exercise Program” or “Exercise Program, Weight-Lifting” or “Exercise Programs, Weight-Lifting” or “Weight Lifting Exercise Program” or “Weight-Lifting Exercise Programs” or

BKCI-SSH, ESCI, CCR-EXPANDED, IC Timespan=Allyears

# 1	**94,569**	**TOPIC:** ("Idiopathic Parkinson's Disease" or "Lewy Body Parkinson's Disease" or "Parkinson's Disease, Idiopathic" or "Parkinson's Disease, Lewy Body" or "Parkinson Disease, Idiopathic" or "Parkinson's Disease" or "Idiopathic Parkinson Disease" or "Lewy Body Parkinson Disease" or "Primary Parkinsonism" or "Parkinsonism, Primary" or "Paralysis Agitans") [illegible]

Clinicaltrials.gov 2022-04-02

Condition: Parkinson Disease / other terms: Random / Intervention: exercise / Study

results: Studies With Results= 21

Condition: Parkinson Disease / other terms: Random / Intervention: Resistance Training / Study results: Studies With Results= 3

Condition: Parkinson Disease / other terms: Random / Intervention: aerobic exercise /

Study results: Studies With Results= 2

Condition: Parkinson Disease / other terms: Random / Intervention: body weight support treadmill / Study results: Studies With Results= 1

Condition: Parkinson Disease / other terms: Random / Intervention: high-speed resistance training / Study results: Studies With Results= 0

Condition: Parkinson Disease / other terms: Random / Intervention: multicomponent exercise program / Study results: Studies With Results= 0

Condition: Parkinson Disease / other terms: Random / Intervention: Strength Training / Study results: Studies With Results= 3

Condition: Parkinson Disease / other terms: Random / Intervention: Nordic Walking /

Study results: Studies With Results= 0

Condition: Parkinson Disease / other terms: Random / Intervention: power training /

Study results: Studies With Results= 0

Condition: Parkinson Disease / other terms: Random / Intervention: treadmill training / Study results: Studies With Results= 4

Condition: Parkinson Disease / other terms: Random / Intervention: walking / Study

results: Studies With Results= 10

Total: 4

1.2 Supplementary 2: References for included studies:

1.3 Canning, C.G., Allen, N.E., Dean, C.M., Goh, L., and Fung, V.S. (2012). Home-based treadmill training for individuals with Parkinson's disease: a randomized controlled pilot trial. Clin Rehabil 26(9), 817-826. doi: 10.1177/0269215511432652.

1.4 Cugusi, L., Solla, P., Serpe, R., Carzedda, T., Piras, L., Oggianu, M., et al. (2015). Effects of a Nordic Walking program on motor and non-motor symptoms, functional performance and body composition in patients with Parkinson's disease. NeuroRehabilitation 37(2), 245-254. doi: 10.3233/nre-151257.

1.5 Demonceau, M., Maquet, D., Jidovtseff, B., Donneau, A.F., Bury, T., Croisier, J.L., et al. (2017). Effects of twelve weeks of aerobic or strength training in addition to standard care in Parkinson's disease: a controlled study. Eur J Phys Rehabil Med 53(2), 184-200. doi: 10.23736/s1973-9087.16.04272-6.

1.6 Dibble, L.E., Foreman, K.B., Addison, O., Marcus, R.L., and LaStayo, P.C. (2015). Exercise and medication effects on persons with Parkinson disease across the domains of disability: a randomized clinical trial. J Neurol Phys Ther 39(2), 85-92. doi: 10.1097/npt.0000000000000086.

1.7 Dibble, L.E., Hale, T.F., Marcus, R.L., Droge, J., Gerber, J.P., and LaStayo, P.C. (2006). High-intensity resistance training amplifies muscle hypertrophy and functional gains in persons with Parkinson's disease. Mov Disord 21(9), 1444-1452. doi: 10.1002/mds.20997.

1.8 Dibble, L.E., Hale, T.F., Marcus, R.L., Gerber, J.P., and LaStayo, P.C. (2009). High intensity eccentric resistance training decreases bradykinesia and improves Quality Of Life in persons with Parkinson's disease: a preliminary study. Parkinsonism Relat Disord 15(10), 752-757. doi: 10.1016/j.parkreldis.2009.04.009.

1.9 Ferreira, R.M., Alves, W., de Lima, T.A., Alves, T.G.G., Alves Filho, P.A.M., Pimentel, C.P., et al. (2018). The effect of resistance training on the anxiety symptoms and quality of life in elderly people with Parkinson's disease: a randomized controlled trial. Arq Neuropsiquiatr 76(8), 499-506. doi: 10.1590/0004-282x20180071.

1.10 Fisher, B.E., Wu, A.D., Salem, G.J., Song, J., Lin, C.H., Yip, J., et al. (2008). The effect of exercise training in improving motor performance and corticomotor excitability in people with early Parkinson's disease. Arch Phys Med Rehabil 89(7), 1221-1229. doi: 10.1016/j.apmr.2008.01.013.

1.11 Frazzitta, G., Maestri, R., Ghilardi, M.F., Riboldazzi, G., Perini, M., Bertotti, G., et al. (2014). Intensive rehabilitation increases BDNF serum levels in parkinsonian patients: a randomized study. Neurorehabil Neural Repair 28(2), 163-168. doi: 10.1177/1545968313508474.

1.12 Kurtais, Y., Kutlay, S., Tur, B.S., Gok, H., and Akbostanci, C. (2008). Does treadmill training improve lower-extremity tasks in Parkinson disease? A randomized controlled trial. Clin J Sport Med 18(3), 289-291. doi: 10.1097/JSM.0b013e318170626d.

1.13 Ni, M., Signorile, J.F., Balachandran, A., and Potiaumpai, M. (2016). Power training induced change in bradykinesia and muscle power in Parkinson's disease. Parkinsonism Relat Disord 23, 37-44. doi: 10.1016/j.parkreldis.2015.11.028.

1.14 Paul, S.S., Canning, C.G., Song, J., Fung, V.S., and Sherrington, C. (2014). Leg muscle power is enhanced by training in people with Parkinson's disease: a randomized controlled trial. Clin Rehabil 28(3), 275-288. doi: 10.1177/0269215513507462.

1.15 Qutubuddin, A., Reis, T., Alramadhani, R., Cifu, D.X., Towne, A., and Carne, W. (2013). Parkinson's disease and forced exercise: a preliminary study. Rehabil Res Pract 2013, 375267. doi: 10.1155/2013/375267.

1.16 Santos, L., Fernandez-Rio, J., Winge, K., Barragán-Pérez, B., González-Gómez, L., Rodríguez-Pérez, V., et al. (2017). Effects of progressive resistance exercise in akinetic-rigid Parkinson's disease patients: a randomized controlled trial. Eur J Phys Rehabil Med 53(5), 651-663. doi: 10.23736/s1973-9087.17.04572-5.

1.17 Schenkman, M., Moore, C.G., Kohrt, W.M., Hall, D.A., Delitto, A., Comella, C.L., et al. (2018). Effect of High-Intensity Treadmill Exercise on Motor Symptoms in Patients With De Novo Parkinson Disease: A Phase 2 Randomized Clinical Trial. JAMA Neurol 75(2), 219-226. doi: 10.1001/jamaneurol.2017.3517.

1.18 Schilling, B.K., Pfeiffer, R.F., Ledoux, M.S., Karlage, R.E., Bloomer, R.J., and Falvo, M.J. (2010). Effects of moderate-volume, high-load lower-body resistance training on strength and function in persons with Parkinson's disease: a pilot study. Parkinsons Dis 2010, 824734. doi: 10.4061/2010/824734.

1.19 Shulman, L.M., Katzel, L.I., Ivey, F.M., Sorkin, J.D., Favors, K., Anderson, K.E., et al. (2013). Randomized clinical trial of 3 types of physical exercise for patients with Parkinson disease. JAMA Neurol 70(2), 183-190. doi: 10.1001/jamaneurol.2013.646.

1.20 van der Kolk, N.M., de Vries, N.M., Penko, A.L., van der Vlugt, M., Mulder, A.A., Post, B., et al. (2018). A remotely supervised home-based aerobic exercise programme is feasible for patients with Parkinson's disease: results of a small randomised feasibility trial. J Neurol Neurosurg Psychiatry 89(9), 1003-1005. doi: 10.1136/jnnp-2017-315728.

1.21 Vieira de Moraes Filho, A., Chaves, S.N., Martins, W.R., Tolentino, G.P., de Cássia Pereira Pinto Homem, R., Landim de Farias, G., et al. (2020). Progressive Resistance Training Improves Bradykinesia, Motor Symptoms and Functional Performance in Patients with Parkinson's Disease. Clin Interv Aging 15, 87-95. doi: 10.2147/cia.S231359.
